# Common mental disorders during the COVID-19 pandemic in Hong Kong: Age-related differences and implications for dementia risk

**DOI:** 10.3389/fpsyt.2022.909162

**Published:** 2022-09-14

**Authors:** Jessie O. T. Kwok, Rachel W. K. Yan, Charlotte P. C. Kwok, Gabriel W. H. Cheng, Cuichan Lin, Brian H. C. Wong, Sheung Tak Cheng, Allen T. C. Lee, Linda C. W. Lam

**Affiliations:** ^1^Department of Psychiatry, The Chinese University of Hong Kong, Hong Kong, Hong Kong SAR, China; ^2^Department of Psychiatry, Tai Po Hospital, Hong Kong, Hong Kong SAR, China; ^3^Department of Health and Physical Education, The Education University of Hong Kong, Hong Kong, Hong Kong SAR, China

**Keywords:** COVID-19, mental health, depression, anxiety, age, dementia

## Abstract

**Background:**

The COVID-19 pandemic has imposed a profound negative impact on the mental health and wellbeing of societies and individuals worldwide. Older adults may be more vulnerable to the mental health effects of the pandemic, either directly from the infection itself or indirectly through the preventive measures. However, the existing literature on mental health in the older age groups has not been consistent so far. The aim of this study was therefore to assess the prevalence of common mental disorders (CMD; including depression and anxiety disorders) given their association with dementia risk, and to further examine age-related differences between older (≥60 years old) and younger (18–59 years old) adult's psychological status during the COVID-19 pandemic.

**Method:**

This was a secondary analysis of a cross-sectional survey-study conducted during the second wave of COVID-19 pandemic in Hong Kong. The survey was disseminated through different social media platforms to the general population and included sociodemographic questions, self-reported physical health, and previous encounter with SARS or COVID-19. CMD was the primary outcome and was assessed using the 6-item Kessler Scale. A total of 1030 adults fulfilled inclusion criteria.

**Results:**

The prevalence of CMD during the pandemic was 16.1%. Compared to younger adults, older adults were significantly less likely to have a CMD (unadjusted OR = 0.07, 95% CI = 0.02–0.30, *p* < 0.001), with 18.1% of younger adults having CMD compared to 1.6% in the older cohort. Age differences remained significant after controlling for sociodemographic factors, physical health, and previous encounter with SARS or COVID-19 (adjusted OR = 0.12, 95% CI = 0.02–0.57, *p* = 0.008).

**Conclusion:**

Common mental disorders are highly prevalent during the COVID-19 pandemic in Hong Kong, though older adults appeared to be less affected mentally. Present findings highlight the urgent need to implement measures and strategies to mitigate the mental health problems, with particular attention to the younger cohort. Given their association with higher dementia risk, early detection and treatment of depression and anxiety disorders will be of critical importance in providing some relief to the already pressurized dementia burden in the longer term.

## Introduction

The outbreak of the COVID-19 pandemic has led to a mental health crisis globally (1, 2). The direct effects of the pandemic (i.e., the coronavirus infection itself) and the secondary impact (i.e., fear of getting infected, fear of death, social distancing, and quarantine) have inevitably created an environment in which many determinants of mental health are affected (3–5). Based on the data published so far, the early stages of pandemic have often found to be associated with increased levels of stress, anxiety, depression, and insomnia in the general population (6–9), with some preliminary findings even suggesting these effects may persist (10) or worsen (11) in the longer term. Such changes in mental health status can have a significant impact on the psychological risk factors that serve as important predictors of dementia (12, 13). Dementia represents one of the most significant public health challenges. Due to the irreversible nature of the disease and the lack of effective treatments, intervention of modifiable risk factors is of great clinical importance in slowing or preventing dementia onset (14). Depression is recognized as one of the modifiable risk factors for dementia (14, 15), whilst other neuropsychiatric symptoms such as anxiety have also been suggested to act as a prodromal symptom (16) as well as a risk factor for cognitive decline (17, 18). With the emerging evidence pointing to detrimental changes in psychological health during the COVID-19 pandemic and that such symptoms are amenable to treatment (19), monitoring the prevalence of common mental disorders (CMD; including depression and anxiety disorders) and identifying vulnerable groups for timely targeted assistance and intervention is therefore a public health priority (20).

Older adults have typically been considered as one of the most vulnerable groups to the consequences of the COVID-19 pandemic (21, 22). This population was not only perceived to be at the greatest risk of severe complications and mortality (23) but also predicted to be more susceptible to the negative psychological impacts of isolation and loss of access to social and health care (24–26). As a response, much concern has risen about the mental health of older adults, and numerous studies have since been conducted. Contrary to expectations however, the postulation that older adult's mental health would be disproportionately affected by the pandemic has not been uniformly supported by the available literature so far. To date, a number of cross-sectional surveys conducted in the first wave of the pandemic have reported that older adults are at most risk for significant deteriorations in their mental health, with elevated levels of anxiety and/or depressive symptoms (27–30). Similar results were also revealed in a study which compared population-based surveys data before and during the pandemic in Hong Kong, elevated rates of stress, anxiety and depression symptoms were prominent during the COVID-19 outbreak, and such increases were particularly evident amongst the older population as compared to the younger population (31). Moreover, a study conducted in China also found the emotional response of older adults aged 60 years and above was more apparent as compared to the other age groups (32).

In contrast, despite all the challenges that the older population may face during the pandemic, lower rates of mental distress in this population compared to younger age groups have been reported, with older adults faring better than younger adults in multiple metrics of mental health (33). Several large representative surveys of adults in the US (34), UK (35), Denmark (36), Slovenia (37) and China (38) have also found an inverse relationship between age and mental health symptoms. Compared to younger adults, older adults seem to respond to the pandemic with a more positive emotional response and reported lower rates of anxiety and depression. To further complicate the findings, however, some studies have even found that mental health symptoms (i.e., the prevalence and severity of depression and anxiety symptoms) were not differentiated based on age (7, 39–41). Such inconsistencies emphasize the need for further research to explore age differences in the psychological impact of COVID-19, whilst taking into consideration of the potential confounding effects of various social determinants of mental health changes during the COVID-19 pandemic such as female, lower socioeconomic status as well as subjective poorer physical health (42–44). Moreover, it is worth noting that most of the studies outlined above (except Pedersen et al. (36) and Prelog et al. (37)) provided only a snapshot of the immediate and the early months of the mental health responses following the COVID-19 outbreak, which may evolve with the development of the pandemic, public health interventions, and repeated exposure to social distancing regulations.

In this context, this study aimed to (i) evaluate the prevalence of CMD during the second wave of the COVID-19 pandemic in Hong Kong, given their high prevalence during the early stages as well as prior to the COVID-19 pandemic (13.3%, see Lam et al. (45)) and (ii) to further examine whether mental health differs amongst the older and younger population in response to the pandemic.

## Methods

### Study setting, design and participants

This study was a secondary analysis of a cross-sectional survey conducted in Hong Kong during the COVID-19 pandemic (46). This planned secondary analysis specifically concerned age differences and prevalence of mental health problems of the same cohort.

Participant recruitment was conducted between 17 June and 31 July 2020, during the outbreak of the second wave of the COVID-19 pandemic in Hong Kong. The survey was disseminated using the university mass email system and various social media platforms (including Facebook, WhatsApp and WeChat) to the general population and the community cohorts from our ongoing government-commissioned studies, where the households were randomly selected based on the addresses from all 18 districts of Hong Kong generated from the Census and Statistics Department of the Government of Hong Kong. Participants were included if they were Hong Kong residents aged 18 years and over with internet access. Those who were younger than 18, non-local residents, or having significant impairments in communication or understanding instructions were excluded. Completion of the whole survey took about 5 min, and participants only needed to choose the answers that best reflected themselves rather than what they hoped they should be. Participants were free to participate or withdraw anytime from the survey, with no negative consequences associated in those who did not complete or submit their responses online. As there was no direct contact with participant or data collection of personal identifiers, informal consent was sought from participants (i.e., those who successfully completed and submitted a response online was deemed as giving their implied consent).

Ethics approval was obtained from the Survey and Behavioral Research Ethics Committee at the University and study registration was completed (ChiCTR 2000033936) before commencement of the survey. This study was performed in accordance with the ethical standards laid down in the 1964 Declaration of Helsinki and its later amendments.

### Assessment of mental health problems

The 6-item Kessler scale (K6) was used in the present study. The K6 is a simple and quick self-administered rating scale developed to assess psychological distress and screen for CMD in the general population (47). The scale has been validated locally with good psychometric properties reported (48). Participants were asked to rate how often they felt (1) nervous, (2) hopeless (3) restless or fidgety, (4) so depressed that nothing could cheer them up, (5) that everything was an effort, and (6) worthless in the last 30 days. Each item codes from 0 to 4, yielding a total K6 score that ranges from 0 to 24. Higher scores are indicative of greater symptom severity. A cut-off score of 13 was used to define CMD, as previously suggested as indicative of severe mental distress (47).

### Assessment of covariates

The following potential confounding factors were examined: basic sociodemographics (sex, educational level, employment status and retirement), self-reported physical health status (assessed by the 5-point Likert scale of self-rated assessment used in the World Health Survey (49), possible choices were “very good,” “good,” “moderate,” “bad” or “very bad”), and previous personal or close encounter with severe acute respiratory syndrome (SARS) in 2003 or COVID-19 (participants, their family members, or people with whom they had close contact diagnosed with SARS or COVID-19 before).

### Statistical analysis

As this was a secondary analysis of an earlier published study, the sample size was predetermined (46). Statistical analysis was performed using the IBM SPSS Statistics, Version-26.0 (IBM Corp). In this study, age was used both as a continuous and binary variable, with a cut-off of 60 years old as a separation between older (≥60 years) and younger adults (18–59 years). First, comparisons of variables between older and younger participants were analyzed using the independent *t*-test or the χ^2^, as appropriate. The level of statistical significance was set at *p* < 0.05 (two-tailed). We analyzed self-rated physical health as a dichotomous measure, with “bad” and “very bad” as poor. To determine the relationship between age (continuous variable) and K6 total score, Spearman's correlation coefficient was used. Where a significant correlation was found, linear regression further analyzed the relationship, first unadjusted and then adjusting for covariates including sex, educational level, employment status, retirement, physical health, and previous encounter with SARS or COVID-19. Finally, logistic regression analysis was employed to examine the association between age (categorical variable) and CMD, with the former treated as the independent variable and the latter as the dependent variable. Model 1 was unadjusted, whereas Model 2 was adjusted for the same potential confounding factors. The odds ratios (ORs) were computed to yield point estimates with 95% confidence intervals (CI). The younger population served as the reference group.

## Results

### Prevalence and characteristics of participants by age group

A total of 1036 individuals responded to the online survey. Of these, six were excluded as they did not meet the inclusion criteria. Hence the final analysis included 1,030 participants. The cohort consisted of 905 younger adults and 125 older adults respectively. Compared to the younger adults, older adults were more likely to have lower educational level, retirement, and a lower K6 total score. 18.1% of younger adults reported having CMD compared to 1.6% in the older cohort. There was no significant difference in sex, unemployment rate, self-perceived physical health, or previous encounter with SARS or COVID-19 between the two groups (Table 1).

**Table 1:** Comparison of characteristics between younger and older adults (*n* = 1,030).

**Characteristics**	**Younger adults (18–59 years) *n* = 905**	**Older adults (≥60 years) *n* = 125**	**P-value**
Female, *n* (%)	634 (70.1)	79 (63.2)	0.12
Tertiary educational level, *n* (%)	663 (73.3)	43 (34.4)	<0.001
Unemployment, *n* (%)	37 (4.1)	5 (4.0)	0.96
Retirement, *n* (%)	14 (1.5)	72 (57.6)	<0.001
Poor physical health, *n* (%)	156 (17.2)	25 (20.0)	0.45
Previous encounter with SARS or COVID-19, *n* (%)	26 (2.9)	5 (4.0)	0.49
K6 total score, mean (SD)	8.1 (4.7)	4.7 (3.3)	<0.001
Common mental disorders (*n*, %)	164 (18.1)	2 (1.6)	<0.001

### Association between age and K6 total score

**Figure 1:**
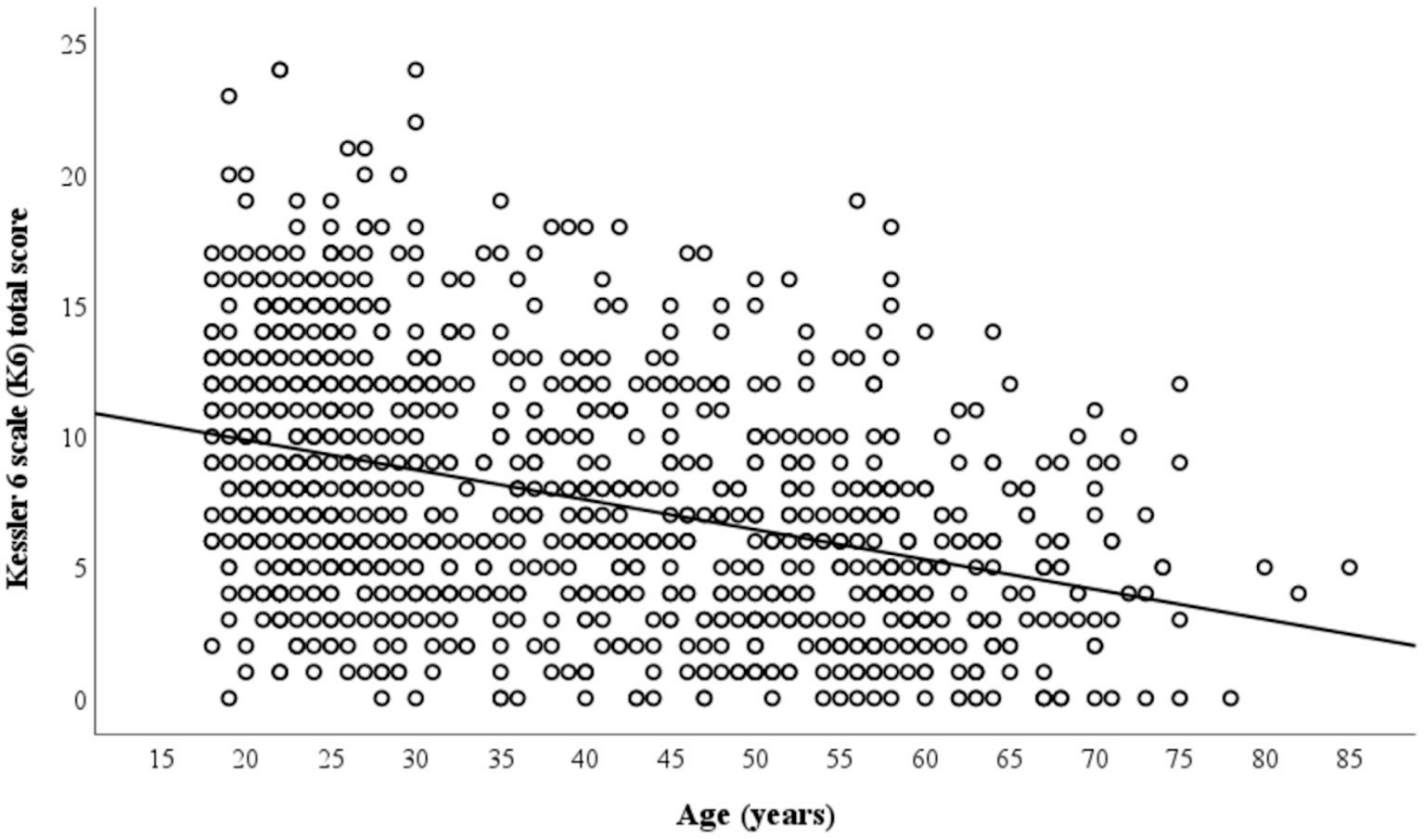
Correlation between participant age and K6 total score.

Correlation analyses revealed a statistically significant inverse relationship between age and total K6 score (*r* = −0.378, *p* < 0.001). The likelihood of having a higher K6 total score, thus poorer mental health appears to decrease with age (Figure 1). Consistent with such findings, linear regression analysis also found that age was associated with K6 total score. For each 1-year increase in age the expected decrease in K6 total score was 0.11 points (*B* = −0.11, 95% CI = −0.13 to −0.10, *p* < 0.001). This association remained significant after adjusting for sex, educational level, employment status, retirement, physical health and previous encounter with SARS or COVID-19 (*B* = −0.13, 95% CI = −0.15 to −0.11, *p* < 0.001; Table 2).

**Table 2:** Linear regression analysis of associations between age and K6 total score.

	***B***	**SE**	**Standard β**	**95% CI**	**P-value**
**Unadjusted regression model** (adjusted *R*^2^ = 0.14)					
Age	−0.11	0.01	−0.38	−0.13 to −0.10	<0.001
**Adjusted regression model** (adjusted *R*^2^ = 0.18)					
Age	−0.13	0.01	−0.42	−0.15 to −0.11	<0.001
Female	0.30	0.29	0.03	−0.28 to 0.87	0.31
Tertiary educational level	−0.70	0.33	−0.07	−1.34 to −0.05	0.04
Unemployment	0.30	0.68	0.01	−1.03 to 1.63	0.66
Retirement	0.08	0.56	0.01	−1.02 to 1.19	0.88
Poor physical health	2.28	0.35	0.18	1.59 to 2.97	<0.001
Previous encounter with SARS or COVID-19	1.07	0.78	0.04	−4.72 to 2.60	0.17

CI, confidence interval; SE, standard error; SARS, severe acute respiratory syndrome.

### Association between age group and CMD

Logistic regression analyses revealed that the odds of reporting CMD was significantly lower in the older adult population than in the younger population (unadjusted OR = 0.07, 95% CI = 0.02 to 0.30, *p* < 0.001). This association remained statistical significance after adjusting for covariates including sex, educational level, employment status, retirement, physical health, and previous encounter with SARS or COVID-19 (adjusted OR = 0.12, 95% CI = 0.02 to 0.57, *p* = 0.008; Table 3). Apart from younger age, poor physical health (adjusted OR = 2.58, 95% CI = 1.74 to 3.82, *p* < 0.001) and higher educational level (adjusted OR = 1.56, 95% CI = 1.03 to 2.36, *p* = 0.04) appeared to independently increase the odds of having CMD during the pandemic.

**Table 3:** Regression analysis of determinants of common mental disorders (CMD).

**Variable**	**Model 1**		**Model 2**	
	**OR (95%CI)**	**P-value**	**OR (95%CI)**	**P-value**
18–59 years (younger adults)	Reference group			
≥60 years (older adults)	0.07 (0.02–0.30)	<0.001	0.12 (0.02–0.57)	0.008
Female	1.33 (0.91–1.93)	0.14	1.33 (0.90–1.97)	0.15
Tertiary education level	1.81 (1.22–2.68)	0.003	1.56 (1.03–2.36)	0.04
Unemployment	1.24 (0.56–2.72)	0.60	1.29 (0.57–2.94)	0.55
Retirement	0.11 (0.03–0.47)	0.003	0.52 (0.10–2.61)	0.43
Poor physical health	2.42 (1.65–3.53)	<0.001	2.58 (1.74–3.82)	<0.001
Previous encounter with SARS or COVID-19	1.54 (0.65–3.64)	0.32	1.74 (0.71–4.28)	0.22

Model 1: unadjusted.

Model 2: adjusted for all factors.

## Discussion

The present study sought to examine the prevalence of CMD and to explore age-related differences in mental health during the COVID-19 pandemic in Hong Kong. Findings from this cross-sectional survey extend previous research (7, 31, 38) and demonstrate a high prevalence of depression and anxiety disorders in the general population, and such symptoms may persist over time, even into the subsequent waves of the pandemic. In this study, we found that 16.1% of our cohort were living with CMD during the second wave of COVID-19 pandemic. This was higher than the prevalence of CMD that we found before the pandemic, which was 13.3% (45). Similar findings have also been documented in studies concerning the Hong Kong population in which the authors also reported a marked elevation of 30.6% (31) and 33.8% (50) in anxiety and depression during the first wave of the pandemic. Whilst these numbers may only provide rough comparison estimates, given the variation in the timeframe and measures used to assess mental health problems, but such consistent, marked increases are worthy of attention. So far, much emphasis has been placed on the precautionary measures and medical resources to minimize the spread of the COVID-19 infection and to reduce mortality, but the psychosocial/mental health consequences associated with these measures and the virus itself have largely been neglected (51). From a clinical perspective, present findings highlight the urgent need to implement and reconsider public mental health measures and responses in order to meet the added demand for mental health services.

The increased prevalence of CMD imposes significant public health implications, and one of the most concerning and debilitating longer-term impact concerns dementia, which already constitutes a public health emergency (52). Depression represents a risk factor for cognitive decline and dementia (53), with studies consistently associating depression (or depressive symptoms) with a more than two-fold increase in dementia risk (54, 55). Additional studies have also shown that depression accelerates the progression and conversion from a cognitively normal state to mild cognitive impairment and dementia (56–58), and those with persistent symptoms exhibit more rapid pathological brain aging (59) and are at greater risk of cognitive decline (15). Moreover, longer durations of untreated depression are correlated with hippocampal atrophy (60) indicating progressive neurodegeneration and dementia is involved in depression symptomatology. Although relatively less studied, recent evidence suggests anxiety may confer an additional risk for incident cognitive decline and dementia (17). Among mid-life and older community adults, increased anxiety was found to predict verbal memory deterioration over a 12-year follow-up period (18). Likewise, midlife anxiety symptoms have been associated with an increased risk for the development of dementia, where the mean interval between anxiety assessment and dementia diagnosis was more than 10 years (61). Adding to the evidence base, a recent meta-analysis also identifies anxiety as associated with a 24% higher risk of developing all-cause dementia (62). More recently, asymmetric atrophy of the hippocampus has also been demonstrated in humans with Alzheimer's disease (AD) and was found to increase with social isolation in a study using animal models for AD (63). More importantly, isolation was associated with an increased and worsening of neuropsychiatric symptoms, and such symptoms may function as the underlying mechanisms responsible for such an association between COVID-19 related isolation and worsening of AD-brain hippocampal asymmetry (63). Taken together the evidence, although mental health effects on dementia incidence was not directly assessed in the present study, the increased prevalence of depression and anxiety disorders during the COVID-19 pandemic has the potential to increase the risk for subsequent dementia, as well as worsen its symptoms. Early identification and timely treatment of this increasing CMD is thus crucial in preventing another public health crisis in the near future and extend the quality of life in old age.

The awareness that increased age is a risk factor for COVID-19-related mortality, together with the restrictions on social interactions inducing loneliness and isolation, had a psychological impact on older adults during the pandemic. However, in this study, we did not observe such an effect amongst the older population. In fact, results showed that age is negatively associated with K6 score. In particular, compared to our cohort of younger adults, older adults were less likely to report poor mental health, with 18.1% of younger adults reported having depression and anxiety disorders, compared to only 1.6% of those in the older population. Importantly, age differences in mental health remained significant after accounting for socioeconomic factors and participants' previous encounter with SARS or COVID-19. The present findings are consistent with existing studies suggesting that older adults may be less negatively affected by COVID-19 related mental health problems, report fewer negative emotions (64), and experience less anxiety and depression compared to their younger counterparts (36, 65). Although research is still ongoing, these studies seem to suggest that at least a sub-population of older adults is emotionally resilient, potentially owing to their complex experiences and adaptive coping skills/strategies built during their previous lives. Indeed, their resilience has been found to be less influenced by stressful life events (66) and associated with meaning in life (67), which may in part explain the better mental health outcomes observed in the older age groups.

Self-perceived physical health was in the lower interests of researchers during the COVID-19 pandemic. Interestingly, in this study we found that physical health status mediated the relationship between age and mental health outcomes, such that older adults who perceived their physical health as poor were more likely to report CMD than their counterparts who perceived their physical health as good. Similar findings have also been reported elsewhere, in which the authors found that anxiety and depressive symptoms were more frequent amongst adults who subjectively assessed their physical health as poor (68). The present findings may be of clinical importance and suggests that better self-perceived health may serve as a relative protecting factor for the impact of the COVID-19 pandemic on mental health but further investigation is warranted before conclusions can be drawn.

### Limitations

Despite its interesting results, the study has important limitations. First, as this was a cross-sectional observational study, it is difficult to elucidate a causal relationship between the COVID-19 pandemic and mental health problems. As the pandemic continues to evolve, continuous efforts are needed to monitor the wellbeing of the general population. Second, the present findings might not be generalizable to other populations. Different countries are characterized by different COVID-19 incidence and death rates, and there are wide variations in governmental restrictions which in turn is likely to have differential impacts on mental health. Third, assessment of mental health problems relied on self-rated measures, and health records were not reviewed, so the diagnosis, onset and duration of depression and anxiety disorders could not be confirmed. Thus, it cannot be ruled out that study results may have reflected, at least partially pre-existing psychiatric disorders/psychological symptoms. Fourth, due to the strict restriction measures in place, the study was conducted through an online survey distributed by the university mass email system and various social media platforms. Recruitment using several platforms was made in an attempt to optimize response rate, however the underrepresentation of older adults in our sample is evident. It must be mentioned that whilst the university mass email system targeted different populations including students, alumni, staff members, as well as retirees, the exact number of people from different age groups in the mass email system was not known. Furthermore, online recruitment potentially skews the participants toward more digitally experienced and skilled users, resulting in a bias toward representativeness especially of the older and vulnerable populations. Lastly, as the current data were collected anonymously through an online survey, participants' responses and identity such as their sociodemographics could not be verified.

## Conclusion

To conclude, the COVID-19 pandemic has lasted and created a significant negative impact on mental health worldwide. Findings from this study suggests the COVID-19 pandemic was associated with an elevated risk for CMD (depression and anxiety disorders) especially for the younger population. Whilst continuous efforts have focused on the preventive measures, present findings highlight the urgent need for implementation of resources to reduce the COVID-19 related mental health problems. Timely identification and treatment of depression and anxiety disorders will be of critical importance, given their association with dementia risk, prompt tackling may thus provide the opportunity to offer some relief to the already pressurized dementia burden in the longer term.

## Data availability statement

The data that support the findings of this study are available from the corresponding authors AL and LL upon reasonable request.

## Ethics statement

Ethics approval was obtained from the Survey and Behavioural Research Ethics Committee at the Chinese University of Hong Kong before commencement of the survey.

## Author contributions

SC, AL, and LL conceived of and designed the study. JK, RY, and CK searched the literature. GC, CL, and BW designed the online questionnaire and collected the data. JK analyzed and interpreted the data and wrote the paper. AL and LL supervised the study, has full access to all the data in the study, and has the final responsibility for the decision to submit for publication. All authors critically edited, revised the work, and read and approved the final manuscript.

## Conflict of interest

The authors declare that the research was conducted in the absence of any commercial or financial relationships that could be construed as a potential conflict of interest.

## Publisher's note

All claims expressed in this article are solely those of the authors and do not necessarily represent those of their affiliated organizations, or those of the publisher, the editors and the reviewers. Any product that may be evaluated in this article, or claim that may be made by its manufacturer, is not guaranteed or endorsed by the publisher.
